# Supplementary material for: The Small RNA Universe of Capitella teleta
Source: Front Mol Biosci. 2022 Feb 25;9:802814. doi: 10.3389/fmolb.2022.802814 (PMC8915122; doi:10.3389/fmolb.2022.802814)
Supplement: Supplementary file 1 [file DataSheet1.ZIP › Supplement/known/CAPTEscaffold_133_10019.pdf]

Provisional ID	:	CAPTEscaffold_133_10019
Score total	:	28167.2
Score for star read(s)	:	3.9
Score for read counts	:	28156.7
Score for mfe	:	1.9
Score for randfold	:	1.6
Score for cons. seed	:	3
Total read count	:	55240
Mature read count	:	54804
Loop read count	:	0
Star read count	:	436

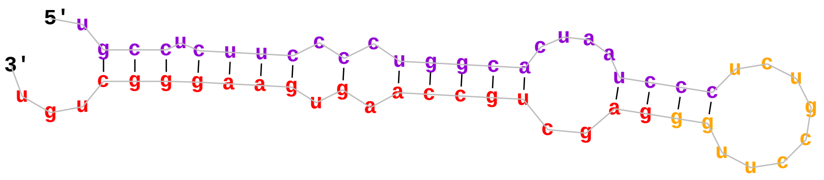

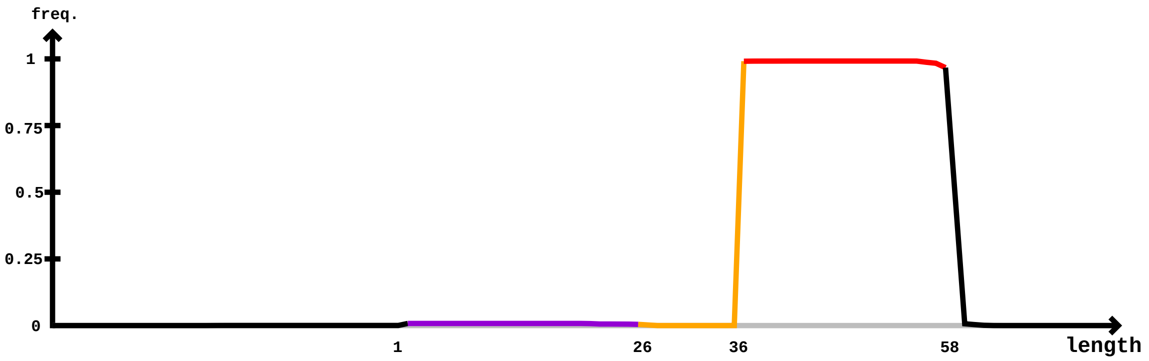


```
                                   Star                              Mature
5'- ugcggcuugggucucccgcugcaugucuggugugugccucuuccccuggcacuaaucccucugccuugggagcugccaagugaagggcuguacaacagcgcaugaccu   -3'   obs
    ugcggcuugggucucccgcugcaugucugguguguugccucuuccccuggcacuaaucccucugccuugggagcugccaagugaagggcuguacaacagcgcaugaccu         exp
    ugcggcuugggucucccgcugcaugucugguguguugccucuuccccuggcacuaaucccucugccuugggagcugccaagugaagggcuguacaacagcgcaugaccu         known
    .(((((..(((...))))))))((((((.((((..(((.((((.(.(((((....((((........))))..))))).).)))))))..)))).))).)))).....     reads   mm   sample
    .....cuCgggucucccgcugcaugucuggugugu..........................................................................     1       1    seq
    ..............cccgcugcaugucuggugugu..........................................................................     1       0    seq
    ...............ccgcugcaugucuggugugu..........................................................................     7       0    seq
    ................cgcugcaugucuggugugu..........................................................................     5       0    seq
    ..................................uugccucuuccccuggcacua......................................................     1       0    seq
    ...................................ugccucuuAcccuggcacu.......................................................     1       1    seq
    ...................................ugccucuuccccuggcacu.......................................................     32      0    seq
    ...................................ugccucuuccccuggcacua......................................................     78      0    seq
    ...................................Agccucuuccccuggcacua......................................................     1       1    seq
    ...................................ugccucuuccccuggcacuaa.....................................................     2       0    seq
    ...................................ugccucuuccccuggcacuaau....................................................     12      0    seq
    ...................................ugccucuuccccuggcacuaauU...................................................     1       1    seq
    ...................................ugccucuuccccuggcacuaauc...................................................     12      0    seq
    ...................................ugccucuuccccuggcacuaaucc..................................................     47      0    seq
    ...................................ugccucuuccccuggcacuaaucGc.................................................     1       1    seq
    ...................................ugccucuuccccuggcacuaauccU.................................................     1       1    seq
    ...................................ugccucuuccccuggcacuaauAcc.................................................     1       1    seq
    ...................................ugccucuuccccuggcacuaGuccc.................................................     1       1    seq
    ...................................Agccucuuccccuggcacuaauccc.................................................     2       1    seq
    ...................................ugccucAuccccuggcacuaauccc.................................................     2       1    seq
    ...................................ugccucuuccccuggcacuaaucAc.................................................     1       1    seq
    ...................................ugccucuuccccuggcacuaauccc.................................................     232     0    seq
    ...................................ugccucuuccccuggcaAuaauccc.................................................     1       1    seq
    ...................................ugccucuuccccuggcacuaaucccu................................................     6       0    seq
    ....................................gccucuuccccuggcacuaauccc.................................................     1       0    seq
    .....................................................................ggagcugccaagugaagggcug..................     4       0    seq
    .....................................................................Cgagcugccaagugaagggcugu.................     1       1    seq
    ......................................................................gagcugccaagugaaggg.....................     1       0    seq
    ......................................................................gGgcugccaagugaagggc....................     2       1    seq
    ......................................................................gagcugccaagugaagAgc....................     1       1    seq
    ......................................................................gagcugccaagugaagggG....................     1       1    seq
    ......................................................................gagcugccaagugaagggU....................     2       1    seq
```

Star Mature

```
ugcggcuugggucucccgcugcaugucugguguguugccucuuccccuggcacuaauccc ucugccuugggagcugccaagugaagggcuguacaacagcgcaugaccu
......................................................................gagcugccaagugaagggc....................   235   0   seq
......................................................................gagcugGcaagugaagggc....................   1   1   seq
......................................................................gagUugccaagugaagggc....................   1   1   seq
......................................................................gagcugAcaagugaagggc....................   2   1   seq
......................................................................gagcugccaGgugaagggc....................   3   1   seq
......................................................................gagcugccaagugaagAgcu...................   1   1   seq
......................................................................gagcugccaaguAaagggcu...................   1   1   seq
......................................................................gagcugccaagugaagggcG...................   1   1   seq
......................................................................gagcugccaagugaagggcA...................   1   1   seq
......................................................................gagcugccaagugaagggcu...................   201   0   seq
......................................................................Uagcugccaagugaagggcu...................   1   1   seq
......................................................................gagcugccaaguAaagggcug..................   2   1   seq
......................................................................gagAugccaagugaagggcug..................   1   1   seq
......................................................................gagcugccaagugaagggcCg..................   1   1   seq
......................................................................gagcugccGagugaagggcug..................   1   1   seq
......................................................................gagcuUccaagugaagggcug..................   1   1   seq
......................................................................gGgcugccaagugaagggcug..................   8   1   seq
......................................................................gagcuCccaagugaagggcug..................   1   1   seq
......................................................................gagcugccaagugaagggcug..................   810   0   seq
......................................................................Aagcugccaagugaagggcug..................   2   1   seq
......................................................................gagcugccaagugaagCgcug..................   1   1   seq
......................................................................gagcugccaagugaagggcGg..................   1   1   seq
......................................................................gagcugccaagCgaagggcug..................   1   1   seq
......................................................................gagcugccaaAugaagggcug..................   59   1   seq
......................................................................gagcugccaGgugaagggcug..................   7   1   seq
......................................................................Uagcugccaagugaagggcug..................   2   1   seq
......................................................................gagcugccaagugaaAggcug..................   2   1   seq
......................................................................gagcugccaagugaagggcuU..................   2   1   seq
......................................................................gagcugccaagGgaagggcugu.................   1   1   seq
......................................................................gagcugccaagAgaagggcugu.................   16   1   seq
......................................................................gagcugccaagugaaggUcugu.................   6   1   seq
......................................................................gagcugccaaguCaagggcugu.................   4   1   seq
......................................................................gagcugccaagugUagggcugu.................   3   1   seq
......................................................................gagcuAccaagugaagggcugu.................   14   1   seq
......................................................................gagcugccaagugaUgggcugu.................   4   1   seq
......................................................................Cagcugccaagugaagggcugu.................   19   1   seq
......................................................................gaUcugccaagugaagggcugu.................   7   1   seq
......................................................................gagcugccaagugaagggcuUu.................   2   1   seq
......................................................................gagcugccaagugaagggcuAu.................   5   1   seq
......................................................................gagcCgccaagugaagggcugu.................   13   1   seq
......................................................................gagAugccaagugaagggcugu.................   5   1   seq
......................................................................gagcugccaGgugaagggcugu.................   192   1   seq
......................................................................gCgcugccaagugaagggcugu.................   2   1   seq
......................................................................gagcugccaagugaaCggcugu.................   2   1   seq
......................................................................gagcugccaagugaagggGugu.................   9   1   seq
......................................................................gagGugccaagugaagggcugu.................   1   1   seq
......................................................................gagcugccaagugaGgggcugu.................   6   1   seq
......................................................................Uagcugccaagugaagggcugu.................   78   1   seq
......................................................................gagcugccaagugaagCgcugu.................   1   1   seq
......................................................................gagcuUccaagugaagggcugu.................   4   1   seq
......................................................................gagcugccaagugaaggCcugu.................   1   1   seq
......................................................................gagcugccaagugaagggcugA.................   52   1   seq
......................................................................gagUugccaagugaagggcugu.................   3   1   seq
......................................................................gagcugccUagugaagggcugu.................   2   1   seq
......................................................................gagcugccaagugaagggcAgu.................   12   1   seq
......................................................................gagcugGcaagugaagggcugu.................   5   1   seq
......................................................................gagcugccaaAugaagggcugu.................   1   1   seq
......................................................................gagcugccaaCugaagggcugu.................   1   1   seq
......................................................................gaAcugccaagugaagggcugu.................   11   1   seq
......................................................................gagcugccaagugGagggcugu.................   3   1   seq
......................................................................gagcugccaagugaagggcCgu.................   3   1   seq
......................................................................gagcugccaagugaagggUugu.................   4   1   seq
......................................................................gagcugccGagugaagggcugu.................   12   1   seq
......................................................................gaCcugccaagugaagggcugu.................   2   1   seq
......................................................................gGgcugccaagugaagggcugu.................   270   1   seq
......................................................................gUgcugccaagugaagggcugu.................   4   1   seq
......................................................................gagcugccaagugaaUggcugu.................   4   1   seq
......................................................................gagcugAcaagugaagggcugu.................   24   1   seq
......................................................................Aagcugccaagugaagggcugu.................   241   1   seq
......................................................................gagcugccaagugaaggAcugu.................   7   1   seq
```

```
                                   Star                               Mature
ugcggcuugggucucccgcugcaugucuggugugugccucuucccccuggcacuaauccc ucugccuugggagcugccaagugaagggcuguacaacagcgcaugaccu
.......................................................................gagcugccaagugaagUgcugu.................  5      1   seq
.......................................................................gagcugccaagCgaagggcugu.................  9      1   seq
.......................................................................gagcugccaaUugaagggcugu.................  3      1   seq
.......................................................................gagcugccaagugaagggcuCu.................  11     1   seq
.......................................................................Nagcugccaagugaagggcugu.................  15     1   seq
.......................................................................gagcAgccaagugaagggcugu.................  26     1   seq
.......................................................................gagcugcUaagugaagggcugu.................  27     1   seq
.......................................................................gagcugccaaguAaagggcugu.................  37     1   seq
.......................................................................gagcugcGaagugaagggcugu.................  1      1   seq
.......................................................................gagcugccaaguUaagggcugu.................  4      1   seq
.......................................................................gagcugccaagugaagggcugC.................  4      1   seq
.......................................................................gagcugccaagugaagggcugu.................  46042  0   seq
.......................................................................gagcuCccaagugaagggcugu.................  2      1   seq
.......................................................................gagcugcAaagugaagggcugu.................  7      1   seq
.......................................................................gagcugccaagugaaAggcugu.................  38     1   seq
.......................................................................gagcugccaagugaagggcugG.................  69     1   seq
.......................................................................gagcugUcaagugaagggcugu.................  8      1   seq
.......................................................................gagcugccaagugaagAgcugu.................  29     1   seq
.......................................................................gagcugccaagugaagggAugu.................  7      1   seq
.......................................................................gagcugccaagugCagggcugu.................  2      1   seq
.......................................................................gagcugccaagugaagggcAgua................  2      1   seq
.......................................................................gagcugccaagugaagCgcugua................  1      1   seq
.......................................................................gGgcugccaagugaagggcugua................  3      1   seq
.......................................................................gagcugccaagugaagggcugAa................  1      1   seq
.......................................................................gagcugccaagAgaagggcugua................  1      1   seq
.......................................................................gagcugccaGgugaagggcugua................  2      1   seq
.......................................................................gagcugccaagugaagggcuguU................  4546   1   seq
.......................................................................Aagcugccaagugaagggcugua................  5      1   seq
.......................................................................Uagcugccaagugaagggcugua................  2      1   seq
.......................................................................gagcugccaagugaagggcuguC................  8      1   seq
.......................................................................gagcugccaagugaagggcugua................  1062   0   seq
.......................................................................gagcugccaagugaagggcuguaG...............  2      1   seq
.......................................................................gagcugccaagugaagggcuguaA...............  78     1   seq
.......................................................................gagcugccaagugaagggcuguac...............  2      0   seq
.......................................................................gagcugccaagugaagggcuguaU...............  88     1   seq
.......................................................................gagcugccaagugaagggcuguaAa..............  140    1   seq
.......................................................................gagcugccaagugaagggcuguaGa..............  1      1   seq
.......................................................................gagcugccaagugaagggcuguacC..............  1      1   seq
.......................................................................gagcugccaagugaagggcuguacU..............  1      1   seq
.......................................................................gagcugccaagugaagggcuguaca..............  2      0   seq
.......................................................................gagcugccaagugaagggcuguaUa..............  1      1   seq
.......................................................................gagcugccaagugaagggcuguaAaa.............  52     1   seq
.......................................................................gagcugccaagugaagggcuguacaac............  1      0   seq
.......................................................................gagcugccaagugaagggcuguaAaac............  2      1   seq
........................................................................Ggcugccaagugaagggcugu.................  1      1   seq
........................................................................agcugccaagugaagggcugu.................  19     0   seq
........................................................................agcugccaagugaUgggcugu.................  1      1   seq
........................................................................agcugccaagugaagggcugua................  1      0   seq
........................................................................agcugccaagugaagggcuguU................  2      1   seq
.........................................................................gcugccaagugaagggcugu.................  5      0   seq
..........................................................................cugccaagugaagggcugu.................  1      0   seq
...........................................................................ugccaagugaagggcugu.................  4      0   seq
...........................................................................ugccaagugaagggcugua................  1      0   seq
............................................................................gccaagugaagggcuguaAaa.............  1      1   seq
```
